# Objective assessment of tumor regression in post-neoadjuvant therapy resections for pancreatic ductal adenocarcinoma: comparison of multiple tumor regression grading systems

**DOI:** 10.1038/s41598-020-74067-z

**Published:** 2020-10-26

**Authors:** Yoko Matsuda, Satoshi Ohkubo, Yuko Nakano-Narusawa, Yuki Fukumura, Kenichi Hirabayashi, Hiroshi Yamaguchi, Yatsuka Sahara, Aya Kawanishi, Shinichiro Takahashi, Tomio Arai, Motohiro Kojima, Mari Mino-Kenudson

**Affiliations:** 1 Oncology Pathology, Department of Pathology and Host-Defense, Faculty of Medicine, Kagawa University, Kagawa, Japan; 2 Department of Pathology, Tokyo Metropolitan Geriatric Hospital and Institute of Gerontology, Tokyo, Japan; 3 Division of Hepatobiliary and Pancreatic Surgery, National Cancer Center Hospital East, Chiba, Japan; 4 Department of Human Pathology, Juntendo University, School of Medicine, Tokyo, Japan; 5 Department of Pathology, Tokai University School of Medicine, Kanagawa, Japan; 6 Department of Anatomic Pathology, Tokyo Medical University, Tokyo, Japan; 7 Department of Gastrointestinal and Pediatric Surgery, Tokyo Medical University, Tokyo, Japan; 8 Department of Gastroenterology and Hepatology, Tokai University School of Medicine, Kanagawa, Japan; 9 Division of Pathology, Research Center for Innovative Oncology, National Cancer Center Hospital East, 6-5-1, Kashiwanoha, Kashiwa-shi, Chiba Japan; 10 Department of Pathology, Massachusetts General Hospital and Harvard Medical School, 55 Fruit Street, Warren 122, Boston, MA USA

**Keywords:** Gastrointestinal cancer, Cancer, Biomarkers

## Abstract

Neoadjuvant therapy is increasingly used to control local tumor spread and micrometastasis of pancreatic ductal adenocarcinoma (PDAC). Pathology assessments of treatment effects might predict patient outcomes after surgery. However, there are conflicting reports regarding the reproducibility and prognostic performance of commonly used tumor regression grading systems, namely College of American Pathologists (CAP) and Evans’ grading system. Further, the M.D. Anderson Cancer Center group (MDA) and the Japan Pancreas Society (JPS) have introduced other grading systems, while we recently proposed a new, simple grading system based on the area of residual tumor (ART). Herein, we aimed to assess and compare the reproducibility and prognostic performance of the modified ART grading system with those of the four grading systems using a multicenter cohort. The study cohort consisted of 97 patients with PDAC who had undergone post-neoadjuvant pancreatectomy at four hospitals. All patients were treated with gemcitabine and S-1 (GS)-based chemotherapies with/without radiation. Two pathologists individually evaluated tumor regression in accordance with the CAP, Evans’, JPS, MDA and ART grading systems, and interobserver concordance was compared between the five systems. The ART grading system was a 5-tiered system based on a number of 40× microscopic fields equivalent to the surface area of the largest ART. Furthermore, the final grades, which were either the concordant grades of the two observers or the majority grades, including those given by the third observer, were correlated with patient outcomes in each system. The interobserver concordance (kappa value) for Evans’, CAP, MDA, JPS and ART grading systems were 0.34, 0.50, 0.65, 0.33, and 0.60, respectively. Univariate analysis showed that higher ART grades were significantly associated with shorter overall survival (p = 0.001) and recurrence-free survival (p = 0.005), while the other grading systems did not show significant association with patient outcomes. The present study revealed that the ART grading system that was designed to be simple and more objective has achieved high concordance and showed a prognostic value; thus it may be most practical for assessing tumor regression in post-neoadjuvant resections for PDAC.

## Introduction

The annual incidence of pancreatic ductal adenocarcinoma (PDAC) has increased worldwide, and PDAC is a major cause of cancer-related death in Europe and the United States^1–3^. In Japan, PDAC is the fifth and third leading cause of cancer-related death in men and women, respectively^4^. Despite improvements in diagnostics and therapeutics, the prognosis of PDAC remains dismal, with a 5-year overall survival (OS) rate of approximately 5%^5,6^. Because of the lack of effective screening methods and the aggressive biology of PDAC, it is typically diagnosed at an advanced stage when patients present with symptoms^7^. Only 15–20% of patients present with resectable disease, while 30% present with borderline resectable or locally advanced disease^8^. In addition, the 5-year OS rate is only 30%, even among those who underwent curative resection^9^. Thus, neoadjuvant therapy (NAC) is increasingly used to control local tumor spread and micrometastasis of PDAC. Recent studies have shown that NAC improves OS in patients with resectable and borderline resectable PDAC^10–14^. Furthermore, a Japanese phase III study demonstrated the significant survival benefits of gemcitabine and S1 after NAC for patients with resectable PDAC^15^. However, prognostic markers for PDAC resected after NAC are yet to be determined.

Pathology assessments of residual tumors and tumor regression may be useful to predict patient outcomes after post-neoadjuvant resections for PDAC. However, multiple systems are currently available to assess tumor regression, and each system has distinct criteria; thus, it is difficult to correlate grades between the systems. The most commonly used tumor regression grading systems worldwide are the College of American Pathologists (CAP) and Evans’ systems. Recently, new systems have been introduced by The University of Texas M.D. Anderson Cancer Center (MDA)^12,16,17^ and the Japan Pancreas Society (JPS) (Table 1)^18^. Both the Evans’ and JPS grading systems are specific for PDAC and are commonly used in Japan^19,20^. The CAP grading system is not specific for PDAC but is also used for other cancers including those of the colon, rectum, and bile duct and is typically used in the United States^21,22^. MDA is similar to CAP, but a three-tiered system instead of four-tiered^12,16^. The Evans’ and JPS grading systems specify a percentage of tumor cell viability or destruction for each grade, whereas the CAP and MDA systems do not. Moreover, the JPS, CAP and MDA grading systems, but not Evans’, require estimating tumor bed (considered to reflect treatment-related fibrosis secondary to tumor cell death) and then evaluating a proportion of the residual tumor. Thus, it is difficult to compare the Evans’, JPS, CAP, and MDA grades (Table 1) except for complete responses (Evans’ IV, CAP 0, MDA 0, and JPS 4) that are easy to understand across all four grading systems.

**Table 1:** The five grading systems used to assess pancreatic tumor regression.

	Definition	Regression criteria
**Evans’ criteria**		
Grade I	**Little (< 10%) or no tumor cell destruction**	**Low**
Grade IIa	**Destruction of 10–50% of tumor cells**	
Grade IIb	Destruction of 51–90% of tumor cells	High
Grade III	Few (< 10%) viable-appearing tumor cells	
Grade IV	No viable tumor cells	
**The College of American Pathologists (CAP)**		
Score 0	No viable cancer cells	High
Score 1	Single cells or small groups of cancer cells	
Score 2	Residual cancer outgrown by fibrosis	
Score 3	**Minimal or no tumors killed or extensive residual cancer**	**Low**
**MD Anderson (MDA)**		
Score 0	No viable tumor cells	High
Score 1	< 5% viable tumor cells	
Score 2	** ≥ 5% viable tumor cells**	**Low**
**The Japanese Pancreas Society (JPS)**		
Grade 1a	**Estimated residual rate ≥ 90%**	**Low**
Grade 1b	**Estimated residual rate ≥ 50 and < 90%**	
Grade 2	Estimated residual rate ≥ 10 and < 50%	High
Grade 3	Estimated residual rate < 10%	
Grade 4	No viable cancer cells	
**Area of residual tumor (ART)**		
Score 0	No remaining viable cancer cells	High
Score 1	Spanning ≤ 1 4 × the objective lens field	
Score 2	Spanning > 1 and ≤ 2 4 × the objective lens fields	
Score 3	Spanning > 2 and ≤ 3 4 × the objective lens fields	
Score 4	**Spanning > 3 4 × the objective lens fields**	**Low**

Evans' criteria further apply IIIM (sizeable pools of mucin) and IVM (acellular pools of mucin). ART represents a number of 40x microsocpic fields equivalent to the maximum area of residual tumor. Bold indicates low-grade regression (as shown in Fig. 3).

Another issue associated with the four systems is the ambiguity of some criteria. For instance, it is difficult to determine the viability of degenerative tumor cells. Further, it can be challenging to distinguish treatment-related necrosis (nonviable tumor cells) from tumor necrosis^17,23^. Furthermore, differentiating treatment-related fibrosis from cancer-associated fibrosis may be complex and subjective given that even treatment-naïve PDAC often exhibits prominent fibrosis—desmoplastic reaction and/or associated chronic pancreatitis^23,24^. Such difficulties in the interpretation may cause interobserver disagreement in the assessment of tumor regression.

Recently, we have reported the prognostic utility of measuring the largest area of residual tumor (ART) with a digital platform in pancreatic, gastric, lung, and rectal cancers^25–27^. ART is designed to be more objective by eliminating the process of estimating the original tumor area. Unfortunately, ART may not be practical because imaging software is needed to measure the residual tumor area. Therefore, a semi-quantitative grading system based on a number of microscopic fields equivalent to ART has been proposed^25^. Such a grading system appears to be more objective than the commonly used grading systems and can be applied in routine pathology practice. In the present study, we assessed and compared the reproducibility and prognostic performance of a modified ART grading system with those of the four grading systems using a multicenter cohort, in the hope of identifying the most clinically relevant grading system to assess tumor regression in post-neoadjuvant resections for PDAC.

## Results

### Clinical features of the study cohort

The study cohort consisted of 97 patients with PDAC (median age: 66 years, Supplementary Table 1). Prior to NAC, most (53%) cases were classified as borderline resectable, followed by resectable (31%), metastatic (9%), and locally advanced (7%). The neoadjuvant regimens were GS only in 55 patients (Supplementary Table 2) and GS with radiation in 42 patients (Supplementary Table 3). At the time of resection, most cases had stage I or II tumors with negative resection margins.

### Histological changes following post-neoadjuvant treatment for PDAC

**Figure 1:**
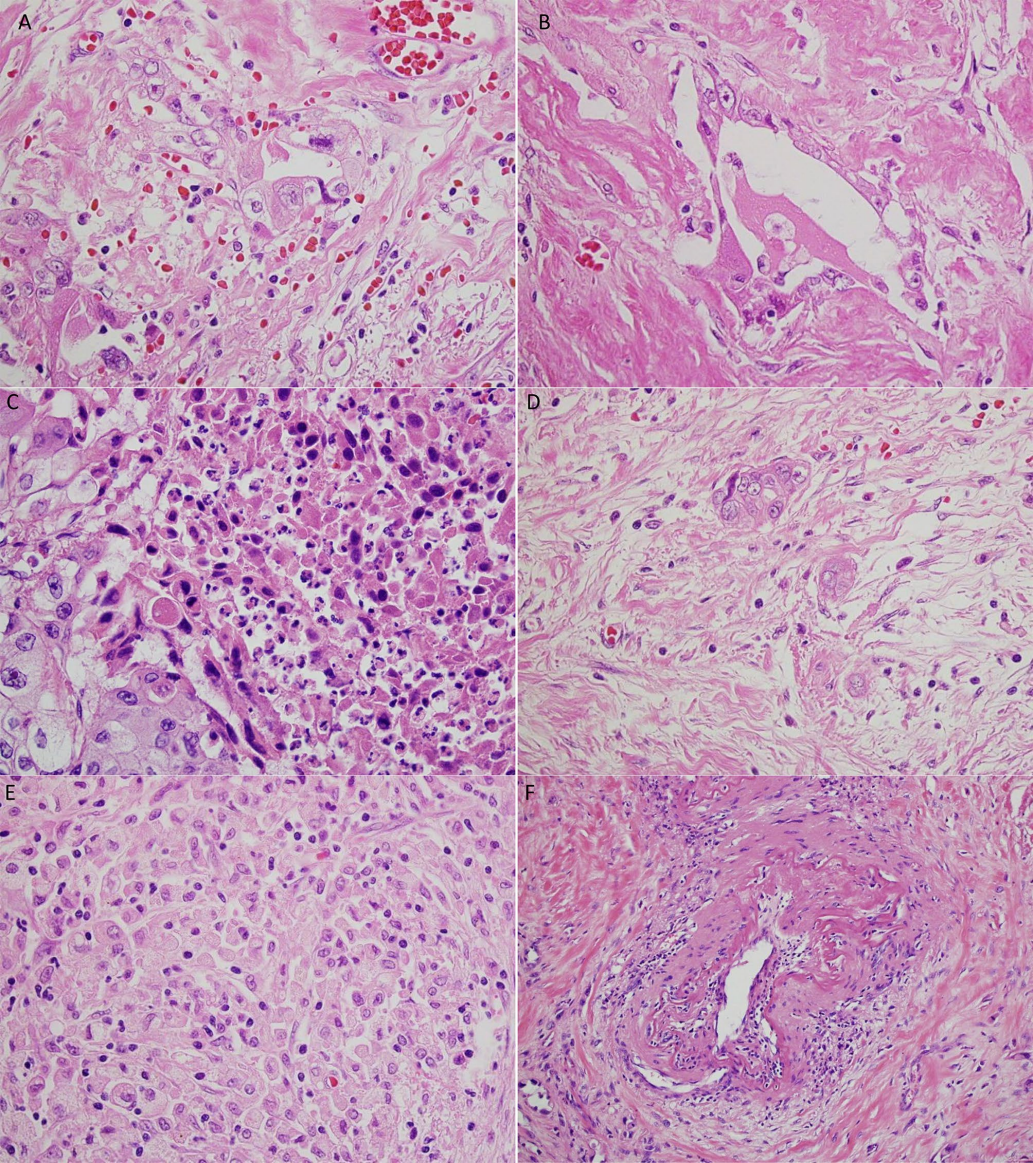
Histologic changes after neoadjuvant treatment for pancreatic ductal adenocarcinoma (PDAC). (**A**) Degenerative cancer cells and inflammatory cell infiltration. (**B**) Degenerative cancer cells in the fibrous tissue. (**C**) Necrotic cancer cells. (**D**) A few cancer cells in the fibrous tissue (major response). (**E**) Macrophage infiltration without cancer cells. (**F**) Degeneration of a vessel.

PDAC following NAC often showed the degeneration (Fig. 1A,B) and necrosis (Fig. 1C) of cancer cells. In this study, we defined non-viable tumor cells as those exhibiting pyknosis, karyorrhexis, karyolysis, or the disappearance of nuclei. If it was difficult to distinguish non-viable cells secondary to treatment from degenerative tumor cells secondary to cancer-related ischemic changes, we did not consider those as non-viable cells to avoid overestimating the treatment effects. As for the assessment of fibrosis, we simply evaluated a ratio of the residual tumor cells over the fibrous stroma for the CAP and JPS grading systems, as it was difficult to differentiate fibrosis secondary to NAC from pre-existing or cancer-related chronic pancreatitis. When a few tumor cells were scattered in the fibrous stroma, it was considered a moderate response based on the fraction of the residual tumor (Fig. 1D). Macrophage aggregates without cancer cells (Fig. 1E), vascular degeneration (Fig. 1F), and acellular mucous pools were also considered treatment effects, and we estimated the total tumor mass before NAC including the areas with those lesions in each case.

### Concordance of tumor regression grading system for PDAC

Table 2 shows the agreement of assessments between the two observers using the five grading systems. The agreement was the highest with the MDA system (95.9%), compared to those with the Evans’, CAP, JPS and ART systems (58.8%, 72.2%, 55.2% and 76.3%, respectively). Among the Evans’, JPS and ART systems with five-tiered grading, ART showed the highest agreement. The interobserver concordance of the five grading systems was fair to substantial (kappa value: Evans’ 0.34, CAP 0.50, MDA 0.65, JPS 0.33 and ART 0.60), and the ART system had the highest value among the 3 five-tiered systems. For individual grades, agreements on Evans’ IIa (41.9%), CAP 1 (42.9%), MDA 1 (42.9%), JPS 2 (30%) and ART 2 (35.3%) were lower than those of the other grades.

**Table 2:** Comparison of tumor regression grades between the two pathologists for the five systems.

Evans’	Kappa = 0.34, percentage of agreement = 58.8%				
	I	IIa	IIb	III	IV
I	**21**	**24**	0	0	0
IIa	**5**	**26**	6	0	0
IIb	0	4	6	0	0
III	0	0	1	3	0
IV	0	0	0	0	1

CAP	Kappa = 0.50, percentage of agreement = 72.2%				
	0	1	2	3	
0	1	0	0	**0**	
1	0	3	2	**0**	
2	0	2	29	**15**	
3	**0**	**0**	**8**	**37**	

MDA	Kappa = 0.65, percentage of agreement = 95.9%				
	0	1	2		
0	1	0	**0**		
1	0	3	**2**		
2	**0**	**2**	**89**		

JPS	Kappa = 0.33, percentage of agreement = 55.2%				
	1a	1b	2	3	4
1a	**15**	**10**	0	0	0
1b	**11**	**26**	3	0	0
2	2	14	9	0	0
3	0	1	2	3	0
4	0	0	0	0	1

ART	Kappa = 0.60, percentage of agreement = 76.3%				
	0	1	2	3	4
0	1	0	0	0	**0**
1	0	7	3	0	**0**
2	0	0	6	4	**4**
3	0	0	0	11	**7**
4	**0**	**0**	**0**	**5**	**49**

Vertical columns were evaluated by pathologist 1 and horizontal rows were evaluated by pathologist 2. Bold indicates low-grade regression (as shown in Fig. 3).

CAP, College of American Pathologists; MDA, MD Anderson; JPS, Japanese Pancreas Society; ART, Area of Residual Tumor.

### Prognostic value of tumor regression grade for PDAC

The median follow-up of the entire study cohort was 20.7 months (range: 0.7–61.7 months). Complete response (Evans’ Grade IV, CAP Score 0, MDA 0, JPS Grade 4 and ART Grade 0) was seen only in one case who showed no recurrence or cancer-specific death. Upon stratifying the study cohort by tumor regression grades in each system, there was a trend toward correlation of lower regression grades with shorter OS and RFS (Supplementary Figures 1 and 2). However, there were significant overlaps in all systems, while the MDA and ART grading systems appeared to have better discrimination in survival curves for both RFS and OS among the five systems.

**Figure 2:**
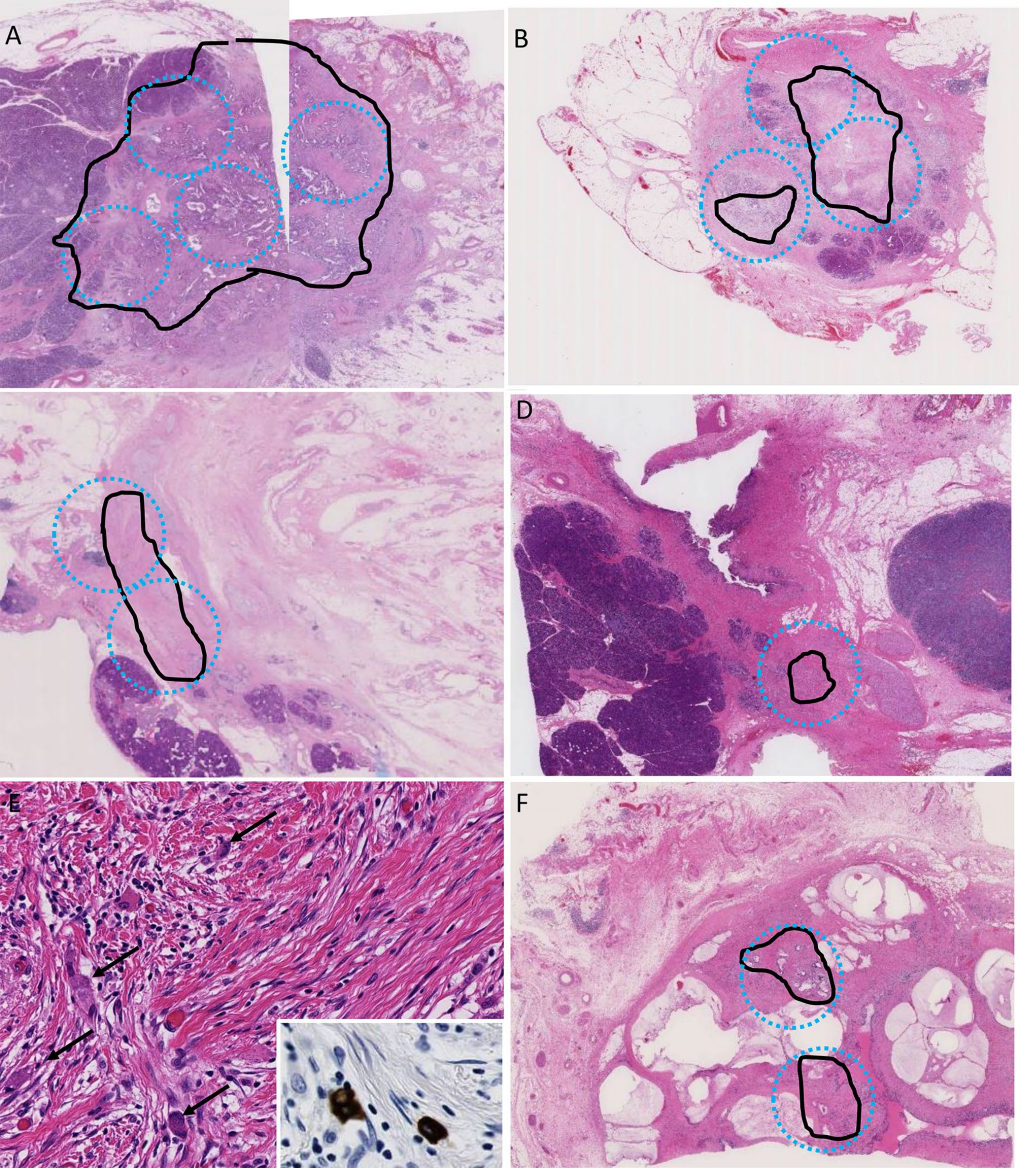
Assessment of the area of residual tumor (ART) scores. (**A**) score 4; (**B**) score 3; (**C**) score 2; (**D**) score 1. (**E**) Enlarged view of (**D**). Arrows indicate cancer cells. Cytokeratin 19 staining is shown in the inset. (**F**) There were two tumor foci at a distance ≥ 2 mm; thus, it was considered score 2. Black line, remnant tumor area; blue circle, estimated view with a ×4 objective lens.

**Figure 3:**
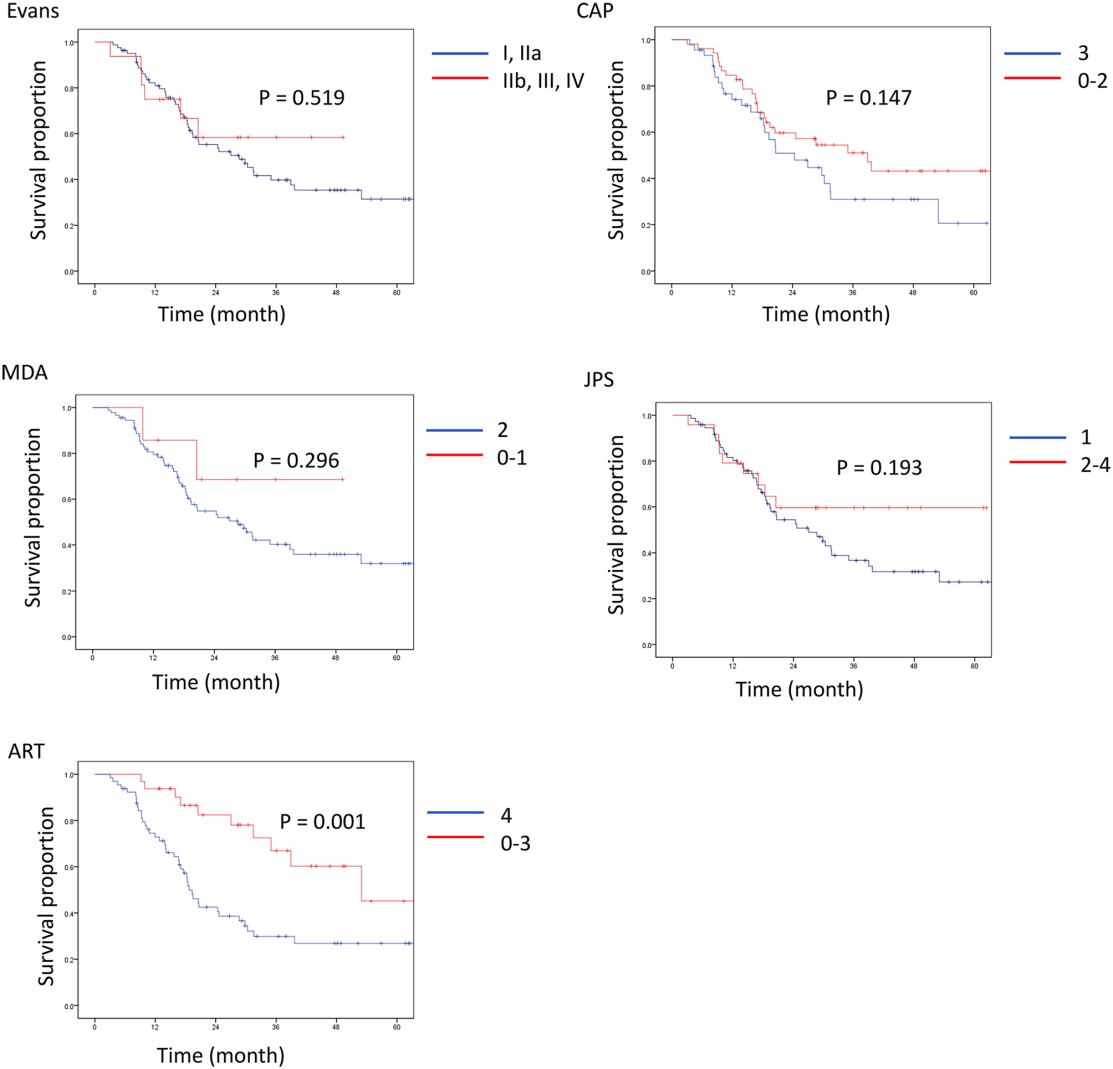
Overall survival after resection stratified by high- vs. low-grade regression. CAP, College of American Pathologists; MDA, the University of Texas M.D. Anderson Cancer Center; JPS, Japan Pancreas Society; ART, Area of Residual Tumor.

**Figure 4:**
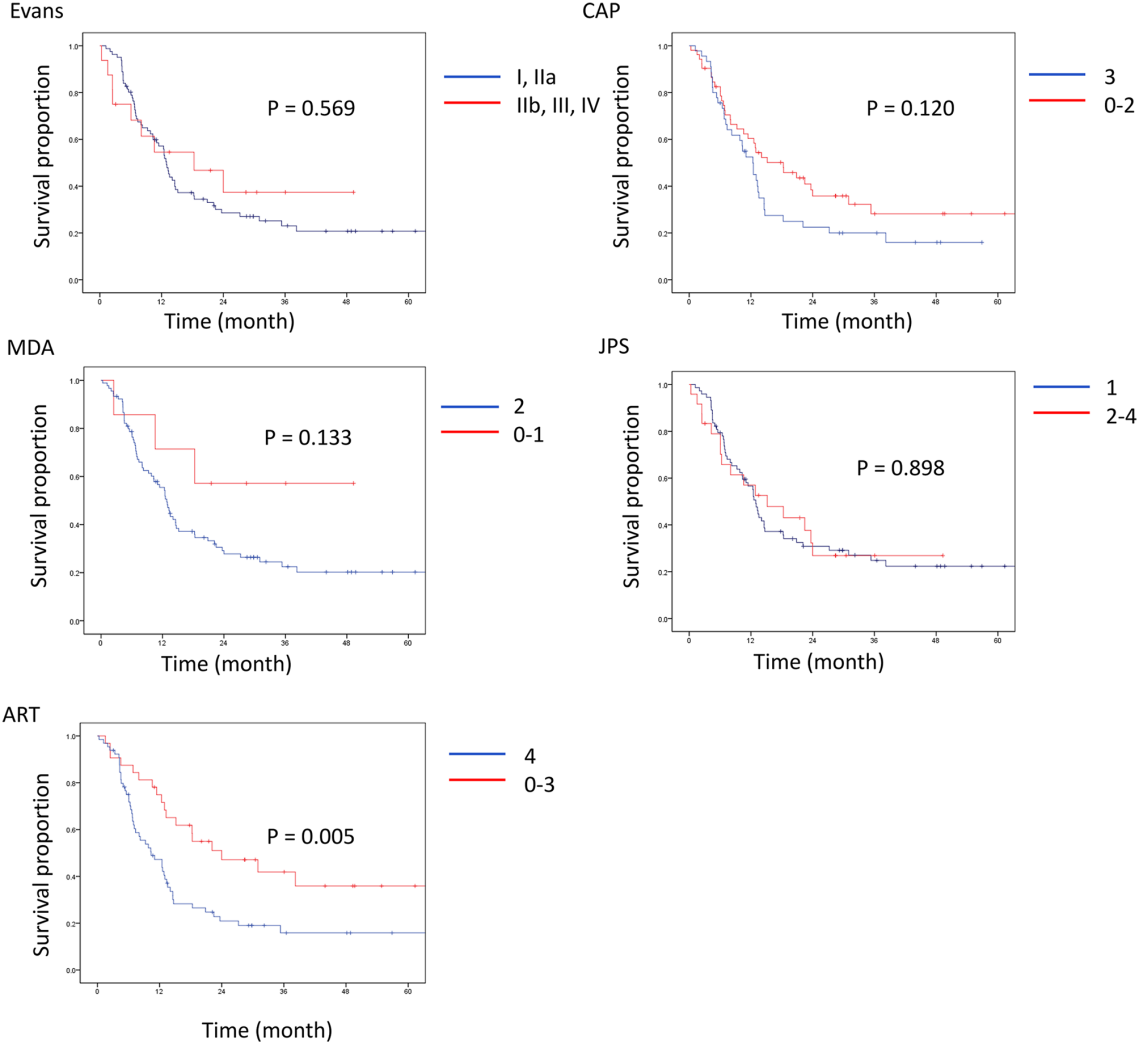
Recurrence free survival after resection stratified by high- vs. low-grade regression. CAP, College of American Pathologists; MDA, the University of Texas M.D. Anderson Cancer Center; JPS, Japan Pancreas Society; ART, Area of Residual Tumor.

Therefore, ROC analysis was performed to determine the best cut-off for high- vs. low-grade tumor regression to predict clinical outcomes in each grading system (Table 3). The analysis identified the cut-off between Grades I and IIa in Evans’, 2 and 3 in CAP, 1 and 2 in MDA, 1 and 2 in JPS and 3 and 4 in ART as the largest areas under the curves, confirming the optimal cut-off point of between 3 and 4 in ART, as proposed in a previous study^25^. Upon using the established cut-off level, univariate analysis showed that the high-grade regression group in the ART grading system had significantly longer OS and RFS than the low-grade regression group, while there was no significant difference in patient outcomes between low- and high-grade regression groups in the other grading systems (Figs. 3, 4 and Supplementary Table 4). However, high-grade regression based on the ART grading system did not remain as a predictor of favorable survival (P = 0.219 for OS and P = 0.253 for RFS) upon multivariate analysis. In this model, small vessel invasion and positive resection margin were associated with shorter OS (P = 0.040 and 0.015, respectively) and the male gender and adjuvant treatment with shorter RFS (P = 0.010 and 0.007, respectively). Furthermore, high-grade tumor regression in accordance with the ART grading system was associated with tumors located in the body and tail, preoperative diagnosis of metastatic disease, negative vascular invasion, negative perineural invasion, and lower pathologic stage in all patients (Table 4), chemotherapy-treated patients (Supplementary Table 2) and chemoradiotherapy-treated patients (Supplementary Table 3).

**Table 3:** Adequate cut-off value to estimate clinical outcomes.

	High-grade regression	Low-grade regression	ROC (for OS)
	Regression grade(s) included/no. of patients	Regression grade(s) included/no. of patients	
Evans', grade/n	I/71	II, III, IV/26	0.527
	**I, IIa/81**	**IIb, III, IV/16**	**0.550**
	I, II/93	III, IV/4	0.523
	I, II, III/96	IV/1	0.511
CAP, grade/n	0/1	1, 2, 3/96	0.511
	0, 1/7	2, 3/90	0.535
	**0, 1, 2/52**	**3/45**	**0.548**
MDA, grade/n	0/1	1, 2/96	0.511
	**0, 1/7**	**2/90**	**0.535**
JPS, grade/n	1b, 2, 3, 4/75	1a/22	0.571
	**2, 3, 4/24**	**1/73**	**0.575**
	3, 4/5	1, 2/92	0.534
	4/1	1, 2, 3/96	0.511
ART, grade/n	0/1	1, 2, 3, 4/96	0.522
	0, 1/9	2, 3, 4/88	0.556
	0, 1, 2/16	3, 4/81	0.591
	**0, 1, 2, 3/32**	**4/65**	**0.641**

The groupings highlighted in bold indicate the final high- and low-grade regression groups determined and used in this study.

ROC, receiver operating characteristic; OS, overall survival; CAP, College of American Pathologists; MDA, MD Anderson; JPS, Japanese Pancreas Society; ART, Area of Residual Tumor.

**Table 4:** Clinicopathological characteristics of high- and low-grade regression groups based on ART scores.

	High-grade regression group (ART score 0, 1, 2, 3)	Low-grade regression group (ART score 4)	P value
No. of patients	32	65	
**Age, years**			
Median (range)	65 (38–84)	68 (49–78)	
≥ 70 (%)	10 (31%)	21 (32%)	0.916
Sex, male (%)	18 (56%)	46 (71%)	0.156
**Tumor location (%)**			
Head/body and tail	15 (47%)/17 (53%)	49 (75%)/16 (25%)	0.005*
**Preoperative diagnosis (%)**			
R/BR/LA/M	8 (25%)/16 (50%)/1 (3%)/7 (22%)	22 (34%)/35 (54%)/6 (9%)/2 (3%)	0.027*
**Preoperative treatment (%)**			
CRT/CT	17 (53%)/15 (47%)	25 (38%)/40 (62%)	0.171
**Tumor differentiation, n (%)**			
G1/G2/G3/others	10 (31%)/13 (41%)/3 (9%)/6 (19%)	32 (49%)/28 (43%)/4 (6%)/1 (2%)	0.013*
**Vascular invasion (%)**			
Negative/positive	18 (56%)/14 (44%)	7 (11%)/58 (89%)	< 0.001*
**Perineural invasion (%)**			
Negative/positive	15 (47%)/17 (53%)	8 (12%)/57 (88%)	< 0.001*
**Stage (UICC 8th) (%)**			
0/IA/IB/IIA	1 (3%)/14 (44%)/8 (25%)/1(3%)	0 (0%)/6 (9%)/19 (29%)/5 (8%)	0.001*
IIB/III/IV	5 (16%)/2 (6%)/1(3%)	24 (37%)/11 (17%)/0 (0%)	
**Negative resection margin, n (%)**	6 (19%)/26(81%)	14 (22%)/51 (78%)	0.750

Gemcitabine- and S-1-based chemotherapies with or without radiation for neoadjuvant treatment.

R, resectable; BR, borderline resectable; LA, locally advanced; M, metastasis; CRT, chemoradiation; CT, chemotherapy; G, histological grade.

*p < 0.05 by chi-square test.

## Discussion

Multiple previous studies have shown the efficacy of NAC for resectable, borderline resectable, and locally advanced PDAC^28^. Volume reduction by NAC has been reported to contribute to the increased number of curative resections with fewer complications and provide better clinical outcomes in PDAC. Pathological features of the tumor after NAC may serve as prognostic factors in these cases. For instance, marked fibrosis, perineural invasion, muscular vessel invasion, and tumor stage as determined by the American Joint Committee for Cancer have been associated with prognosis^12,13,24,29^. In addition, the extent of tumor regression after NAC has been reported as a predictor of clinical outcomes after resection^20,30,31^; thus, it is important to establish a pathology grading system to assess the extent of tumor regression that is clinically relevant and practical. Currently, there are multiple tumor regression grading systems available for post-neoadjuvant pancreatic resections, and few studies have compared the clinical relevance and practicality between those systems^32^. The present study is the first multicenter study to evaluate and compare the reproducibility and prognostic performance among multiple tumor regression grading systems. Of the grading systems evaluated in this study, ART, a new grading system that we had proposed, showed high interobserver concordance and a significant association with patient outcomes in the univariate analysis.

Marked tumor regression greater than Evans’ Grade IIb, in which > 50% of tumor cells are non-viable, has been reported to predict favorable outcomes after resection in patients with PDAC who had received preoperative chemoradiation therapy^33^. Similarly, CAP and MDA Grades 0 & 1 were associated with significantly more favorable patient outcomes than Grades 2 & 3 and Grade 2, respectively^16,20^. In the current study, high-grade tumor regression was associated with better prognosis in all grading systems evaluated, but only the ART grading system showed statistical significance supporting its clinical relevance. This system was based on our prior study that used morphometry to measure ART and showed its significant association with patient outcomes^25^. We established a tumor regression grading system based on a number of 40× microscopic fields equivalent to ART, explored multiple cut-off values to identify the best cut-off, and confirmed the prognostic relevance of the ART grading system in this study, thereby, translating our scientific evidence into pathological practice.

On a somber note, only one (1.0%) patient achieved complete response in this study, significantly less than that previously reported^14,20^. The lower response rate could be attributed to the study cohort comprised of 80% borderline resectable or more advanced tumors, but it may also be explained by the difference in NAC regimens used. For instance, previous studies wherein most patients with PDAC were treated with chemoradiation reported complete response in 2.5–2.7% patients of the study cohort^14,20^. In the current study with the vast majority of patients treated with GS-based chemotherapies, major response was seen in 9.5% of patients who had also received radiation and in 5.5% of those who had received chemotherapy only (P = 0.141, data not shown). These results are consistent with the findings of a recent study in which preoperative chemoradiation therapies resulted in more prominent fibrosis and smaller ART than preoperative chemotherapies for rectal cancer^26^. Furthermore, in a study using preoperative FOLFIRINOX for PDAC, complete response was reported in 13% of the cohort patients^34^. Therefore, large-scale multi-cohort studies are warranted to assess the performance of various NAC regimens on tumor regression.

Reproducibility is a major problem in pathologic assessments for PDAC after NAC. Kalimuthu and colleagues have reported that the CAP, Evans’, and MDA tumor regression grading systems had suboptimal concordance among four gastrointestinal pathologists^17^. Similarly, each of the five systems evaluated in the current study showed fair to moderate concordance between the two observers. The concordance was particularly low with the Evans’ and JPS grading systems that require estimating the degree of tumor cell degeneration, although we defined the viability of tumor cells as precisely as possible. Furthermore, using fibrosis as a surrogate for the pre-treatment tumor area to assess regression could be contentious given the complexity and subjectivity in differentiating fibrosis secondary to treatment from the fibrosis of chronic pancreatitis that is cancer-related and/or pre-existing^17^. While the MDA system with 3 tiered grading that also involves the assessment of tumor bed achieved substantial concordance in this study, we believe that assessing tumor regression based on fibrosis remains controversial. Conversely, the ART grading system does not require the pathologist to estimate the tumor area before NAC and is much simpler than the other systems leading to better reproducibility than the vast majority of the commonly used grading systems. Now, we have shown that the ART grading system has not only prognostic relevance but also good reproducibility; thus, it may be most practical for the assessment of tumor regression in post-neoadjuvant resections for PDAC.

The present study has several limitations. First of all, interobserver concordance was assessed by only two observers. More importantly, the study cohort was relatively small, and most patients were treated with GS-based chemotherapy with or without radiation. Given the efficacy of FOLFIRINOX^34^, an increasing number of patients are being treated with the regimen; thus, we have planned a validation study to evaluate the reproducibility and prognostic utility of the ART grading system by a larger number of observers in larger cohorts that include patients treated with FOLFIRINOX. In addition, multivariate analysis failed to confirm the prognostic significance of ART. It may be attributed in part to the small cohort size, but it also indicates the limitation of prognostic prediction based solely on a single pathological parameter. A comprehensive prediction model with multiple pathological and clinical variables may be more useful to accurately predict and stratify patient outcomes^35^.

In conclusion, the commonly used tumor regression grading systems showed no bearing on patient outcomes after post-neoadjuvant resections for PDAC with fair to substantial interobserver concordance, while the ART grading system that was designed to be simple and more objective has achieved good reproducibility and showed a prognostic value. Although additional studies are warranted to further evaluate the clinical utility of the ART grading system in larger cohorts treated with various neoadjuvant regimens, we believe that it has the potential to become the standard grading system to assess tumor regression in post-neoadjuvant resections for PDAC.

## Materials and methods

### Study cohort

The study cohort consisted of 97 patients with PDAC who had undergone post-neoadjuvant pancreatectomy at the National Cancer Center Hospital East, Juntendo University, Tokai University School of Medicine, or Tokyo Medical University between 2013 and 2017. All patients received gemcitabine and S-1 (GS)-based neoadjuvant chemotherapies with or without radiation (Supplementary Tables 1, 2, and 3). The resected pancreas was routinely fixed with formalin and sectioned every 5 mm vertical to the main pancreatic duct. All sections from the entire pancreatic specimen and lymph nodes were processed in paraffin-embedded tissue blocks (mean, 32 raging from 11 to 66). All tissue blocks were sectioned, stained with hematoxylin and eosin, and microscopically evaluated. The pathological diagnosis for each case was assigned by a gastrointestinal pathologist in accordance with the 2019 WHO Classification of Tumours of the Digestive System^36, 37^. Only patients with conventional PDAC were included in the study cohort, while those with invasive carcinomas arising in association with intraductal papillary mucinous neoplasm or mucinous cystic neoplasm, acinar cell carcinoma or neuroendocrine carcinomas were excluded.

This study was conducted in accordance with the principles embodied in the 2008 Declaration of Helsinki and was approved by the ethics committees of Tokyo Metropolitan Geriatric Hospital (permit #16-47), National Cancer Center Hospital East (#2017-358), Juntendo University (#19-056), Tokai University School of Medicine (#16R273), and Tokyo Medical University (#T2018-0001). Informed written consent to use the tissues was obtained from all patients.

### Evaluation of tumor regression by Evans, CAP, MDA, JPS and ART grading systems

Two observers (Y.M. and M.M.-K.) individually reviewed all histology sections from each case to assess the residual tumor using the following grading systems: (1) Evans’, which evaluates the fraction of necrotic cells among the residual cancer cells; (2) CAP, which evaluates the amount of residual tumor in correlation with fibrosis; (3) MDA, which is a modified CAP system with three-tiered grading^16,17^; (4) JPS, which combines the CAP and Evans’ systems; and (5) the ART grading system (Table 1). The ART system was proposed on the basis of our previous report on ART^26^. We microscopically evaluated the area of residual tumor (black line, Fig. 2A–D) in the slice (cross section) that had the most abundant residual tumor, and then graded tumor regression in accordance with a number of microscopic fields (blue circle) with a 4× objective lens (UPlanSApo 4× , Olympus, Tokyo, Japan; the estimated surface area of 23.75 mm^2^/40× magnification) that collectively cover the largest ART as follows: Score 0, no remaining viable cancer cells; Score 1, ≤ 1 field; Score 2, > 1 and ≤ 2 fields; Score 3, > 2 and ≤ 3 fields; Score 4, > 3 fields (Table 1). When it was difficult to identify the cancer cells with a 4 × objective lens, the specimen with evaluated with higher magnifications. After mapping the ART with higher magnification, we evaluated the ART score with a 4× objective lens. One case required cytokeratin 19 staining to identify the small number of remnant cancer cells (Fig. 2E, arrows and inset indicate cytokeratin 19-positive cancer cells). When multiple residual tumor foci were identified in sections made from the slice and were at least 2-mm apart, we evaluated the individual foci and summed the numbers of microscopic fields (Fig. 2F, score 2). When multiple small foci were present close to each other (within 2-mm), they were considered to form one singe ART. Carcinoma in situ, acellular mucin (Fig. 2F), and lymph node metastasis were excluded from the assessment.

When the two pathologists recorded the same grade, it was used as the final grade. If the two pathologists recorded different grades, the third pathologist (M.K.) reviewed the case to determine the majority grade as the final grade. All observers were surgical pathologists with expertise in the field of PDAC and were blinded to clinical information, the original pathology diagnosis, and the other reviewers’ grades. Interobserver concordance between the two observers (Y.M. and M.M.-K.) was compared among the four systems. Recurrence-free survival (RFS) and OS were correlated with the final grades in each system.

### Statistical analysis

Concordance between the two observers was assessed using Cohen's kappa coefficient. Receiver operating characteristics (ROC) were analyzed to determine the best cut-off value for each grading system. OS and RFS were analyzed based on Kaplan–Meier survival estimates. Significant survival-related factors according to univariate analysis (P < 0.05) were entered in a multivariate Cox proportional-hazards model. Clinicopathological characteristics were analyzed using chi-square test. P < 0.05 was considered to indicate significance in all analyses. Statistical analyses were performed using the StatView J version 5.0 software package (SAS Institute, Inc., Cary, NC, USA) and SPSS version 22 (IBM Corp., New York, NY, USA).

## Supplementary information

Supplementary Figure Legends.

Supplementary Information 1.

Supplementary Information 2.
