# Supplementary material for: Objective assessment of tumor regression in post-neoadjuvant therapy resections for pancreatic ductal adenocarcinoma: comparison of multiple tumor regression grading systems
Source: Sci Rep. 2020 Oct 26;10:18278. doi: 10.1038/s41598-020-74067-z (PMC7588464; doi:10.1038/s41598-020-74067-z)
Supplement: Supplementary file 1 — Supplementary Figure Legends. [file 41598_2020_74067_MOESM1_ESM.docx]

**Supporting information**

**Supplementary Figure 1. Overall survival after resection stratified by tumor regression grades.** CAP, College of American Pathologists; MDA, the University of Texas M.D. Anderson Cancer Center; JPS, Japan Pancreas Society; ART, area of residual tumor.

**Supplementary Figure 2. Recurrence free survival after resection stratified by tumor regression grades.** CAP, College of American Pathologists; MDA, the University of Texas M.D. Anderson Cancer Center; JPS, Japan Pancreas Society; ART, area of residual tumor.
