# Supplementary material for: Objective assessment of tumor regression in post-neoadjuvant therapy resections for pancreatic ductal adenocarcinoma: comparison of multiple tumor regression grading systems
Source: Sci Rep. 2020 Oct 26;10:18278. doi: 10.1038/s41598-020-74067-z (PMC7588464; doi:10.1038/s41598-020-74067-z)

Objective Assessment of Tumor Regression in Post-Neoadjuvant Therapy Resections for Pancreatic Ductal Adenocarcinoma: Comparison of Multiple Tumor Regression Grading Systems

Yoko Matsuda, Satoshi Ohkubo, Yuko Narusawa, Yuki Fukumura, Kenichi Hirabayashi, Hiroshi Yamaguchi, Yatsuka Sahara, Aya Kawanishi, Shinichiro Takahashi, Tomio Arai, Motohiro Kojima, Mari Mino-Kenudson

Supplementary Fig 1

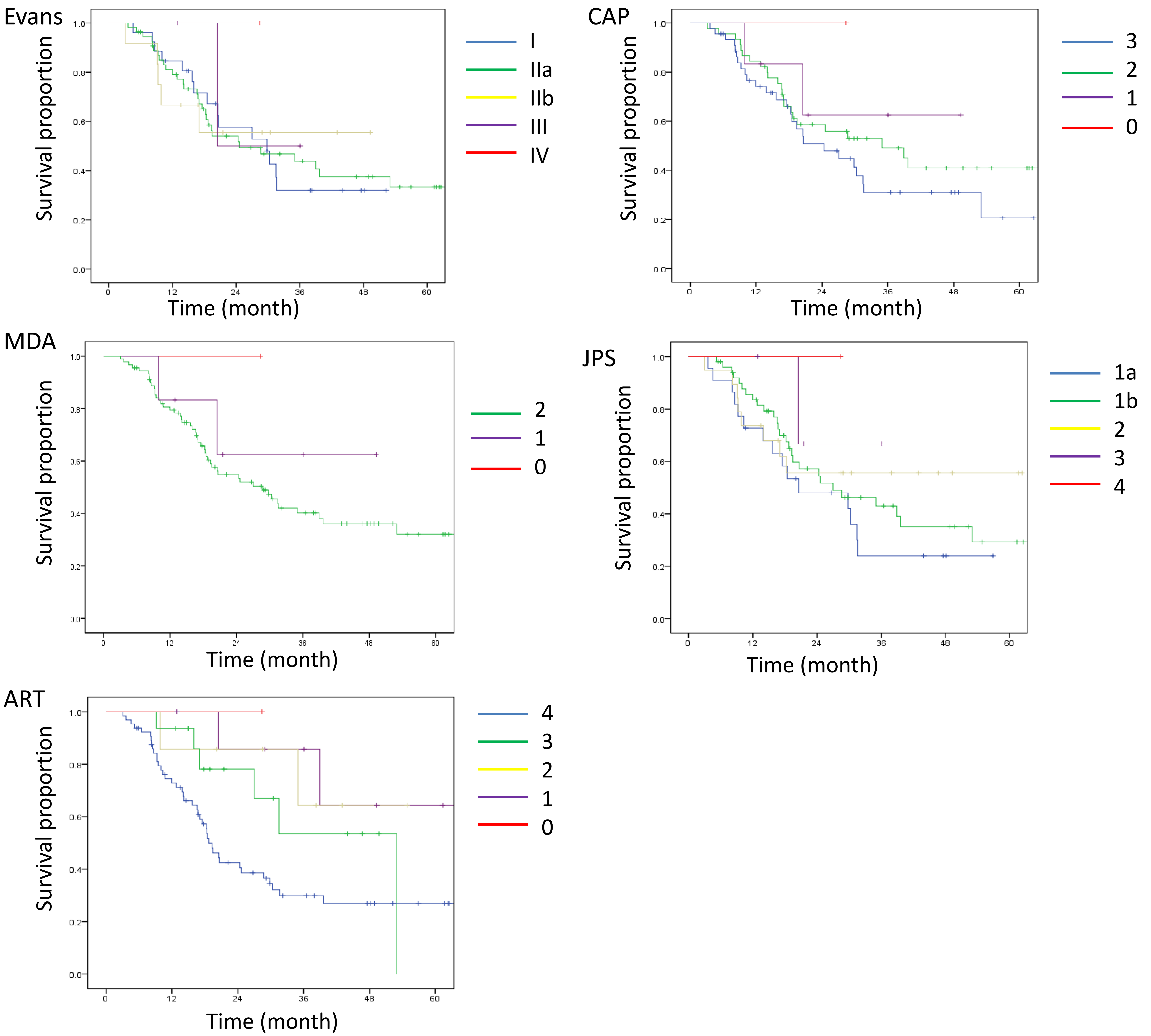

Objective Assessment of Tumor Regression in Post-Neoadjuvant Therapy Resections for Pancreatic Ductal Adenocarcinoma: Comparison of Multiple Tumor Regression Grading Systems

Yoko Matsuda, Satoshi Ohkubo, Yuko Narusawa, Yuki Fukumura, Kenichi Hirabayashi, Hiroshi Yamaguchi, Yatsuka Sahara, Aya Kawanishi, Shinichiro Takahashi, Tomio Arai, Motohiro Kojima, Mari Mino-Kenudson

Supplementary Fig 2

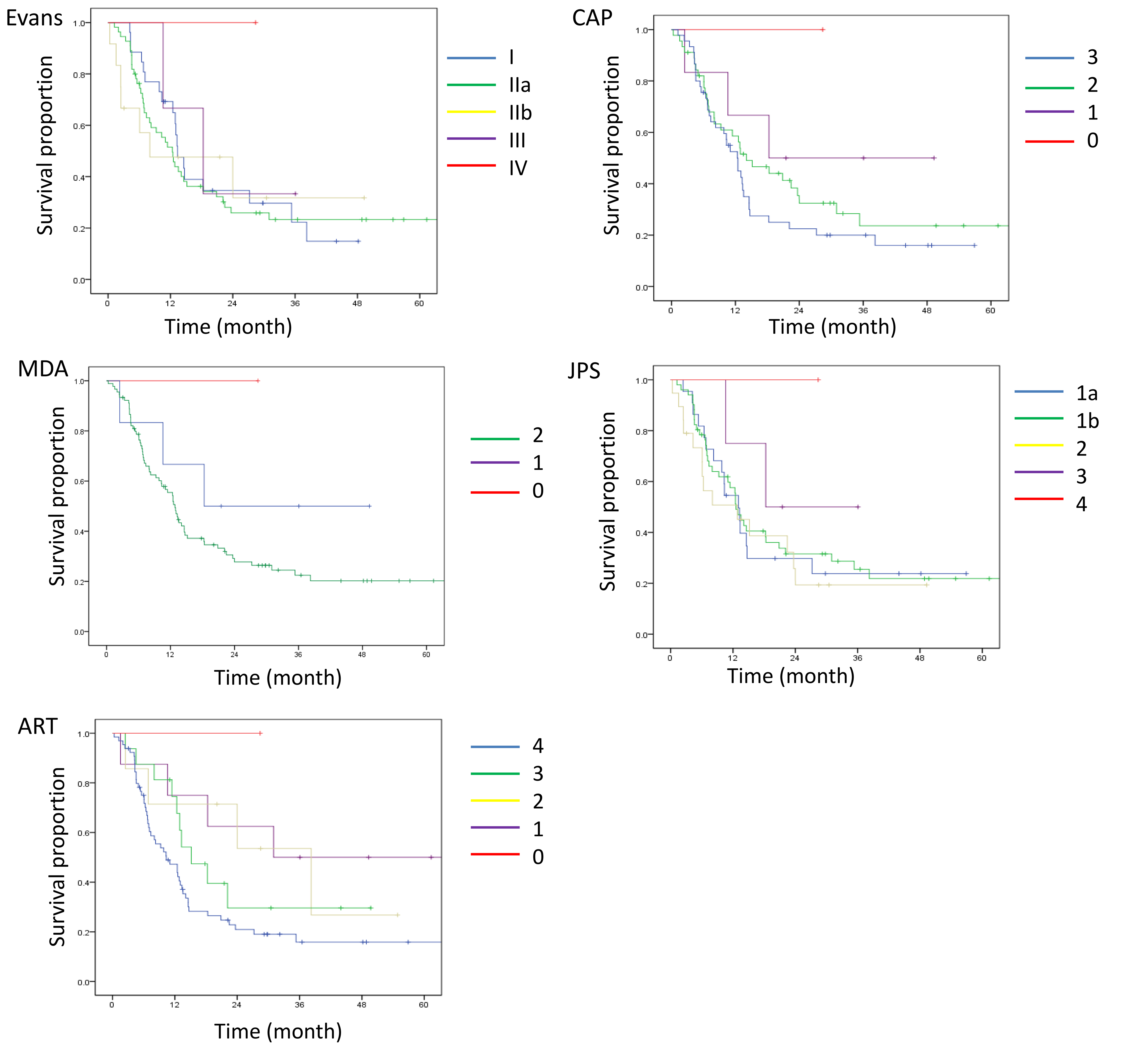

Supplement: Supplementary file 2 — Supplementary Information 1. [file 41598_2020_74067_MOESM2_ESM.pdf]
