# Supplementary material for: Objective assessment of tumor regression in post-neoadjuvant therapy resections for pancreatic ductal adenocarcinoma: comparison of multiple tumor regression grading systems
Source: Sci Rep. 2020 Oct 26;10:18278. doi: 10.1038/s41598-020-74067-z (PMC7588464; doi:10.1038/s41598-020-74067-z)
Supplement: Supplementary file 3 — Supplementary Information 2. [file 41598_2020_74067_MOESM3_ESM.docx]

**Objective Assessment of Tumor Regression in Post-Neoadjuvant Therapy Resections for Pancreatic Ductal Adenocarcinoma: Comparison of Multiple Tumor Regression Grading Systems**

Yoko Matsuda, Satoshi Ohkubo, Yuko Narusawa, Yuki Fukumura, Kenichi Hirabayashi, Hiroshi Yamaguchi, Yatsuka Sahara, Aya Kawanishi, Shinichiro Takahashi, Tomio Arai, Motohiro Kojima, Mari Mino-Kenudson

| Supplementary Table 1. Clinicopathological characteristics of patients with pancreatic cancer who underwent neoadjuvant therapy | | | |  |
| --- | --- | --- | --- | --- |
|  | Total | Chemotherapy | Chemoradiotherapy | P value |
| No. of patients | 97 | 55 | 42 |  |
| Age, years |  |  |  |  |
| Median (range) | 66 (38 - 84) | 66 (38-84) | 68 (44-78) |  |
| ≥70, n (%) | 31 (34%) | 13 (24%) | 18 (43%) | 0.044* |
| Sex, male, n (%) | 64 (60%) | 36 (65%) | 28 (67%) | 0.901 |
| Tumor location, n (%) |  |  |  |  |
| head/ body and tail | 64 (75%)/ 33 (24%) | 40 (73%)/15 (27%) | 24 (57%)/18 (43%) | 0.108 |
| Preoperative diagnosis, n (%) |  |  |  |  |
| R/ BR/ LA/ M | 30 (19%)/ 51 (54%)/ 7 (21%)/ 9 (6%) | 27 (49%)/16 (29%)/4 (7%)/8 (15%) | 3 (7%)/35 (83%)/3 (7%)/1 (2%) | < 0.001* |
| Tumor differentiation, n (%) |  |  |  |  |
| G1/ G2/ G3/ others | 42 (29%)/ 41 (54%)/ 7 (10%)/ 7 (8%) | 22 (40%)/25 (45%)/4 (7%)/4 (7%) | 20 (48%)/16 (38%)/3 (7%)/3 (7%) | 0.626 |
| Vascular invasion, n (%) |  |  |  |  |
| Negative/ Positive | 25 (35%)/ 72 (65%) | 10 (18%)/45 (82%) | 15 (36%)/27 (64%) | 0.050 |
| Perineural invasion, n (%) |  |  |  |  |
| Negative/ Positive | 23 (22%)/ 74(78%) | 11 (20%)/44 (80%) | 12 (29%)/30 (71%) | 0.325 |
| Stage (UICC 8th), n (%) |  |  |  |  |
| 0 | 1 (1%) | 1 (2%) | 0 (0%) | 0.042* |
| IA | 20 (21%) | 9 (16%) | 11 (26%) |  |
| IB | 27 (28%) | 10 (18%) | 17 (40%) |  |
| IIA | 6 (6%) | 5 (9%) | 1 (2%) |  |
| IIB | 29 (30%) | 18 (33%) | 11 (26%) |  |
| III | 13 (13%) | 11 (20%) | 2 (5%) |  |
| IV | 1 (1%) | 1 (2%) | 0 (0%) |  |
| Negative resection margin , n (%) | 77 (86%) | 44 (80%) | 34 (81%) | 0.907 |
| R, resectable; BR, borderline resectable; LA, locally advanced; M, metastatic; G, histological grade. Gemcitabine- and S-1-based chemotherapies with or without radiation for neoadjuvant treatment. *p<0.05 by chi-square test between chemoradiotherapy and chemotherapy groups. | | | | |

| Supplementary Table 2. High- and low-grade regression groups based on ART scores in 55 chemotherapy patients | | | |
| --- | --- | --- | --- |
|  | High-grade regression group  (ART score 0,1,2,3) | Low-grade regression group  (ART score 4) | P value |
| No. of patients | 15 | 40 |  |
| Age, years |  |  |  |
| Median (range) | 61 (38-84) | 68 (49-76) |  |
| ≥70 (%) | 3 (20%) | 10 (25%) | 0.698 |
| Sex, male (%) | 8 (53%) | 28 (70%) | 0.247 |
| Tumor location (%) |  |  |  |
| head/ body and tail | 7 (47%)/ 8 (53%) | 33 (83%)/ 7 (18%) | 0.008* |
| Preoperative diagnosis (%) |  |  |  |
| R/ BR/ LA/ M | 6 (40%)/ 2 (13%)/ 1 (7%)/ 6 (40%) | 21 (53%)/ 14 (35%)/ 3 (8%)/ 2 (5%) | 0.010* |
| Tumor differentiation, n (%) |  |  |  |
| G1/ G2/ G3/ others | 2 (13%)/ 8 (53%)/2 (13%)/ 3 (20%) | 20 (50%)/17 (43%)/2 (23%)/1 (7%) | 0.023* |
| Vascular invasion (%) |  |  |  |
| Negative/ Positive | 7 (47%)/ 8 (53%) | 3 (8%)/ 37 (93%) | 0.001* |
| Perineural invasion (%) |  |  |  |
| Negative/ Positive | 7 (47%)/ 8 (53%) | 4 (10%)/ 36 (90%) | 0.003* |
| Stage (UICC 8th) (%) |  |  |  |
| 0 | 1 (2%) | 0 (0%) | 0.008 |
| IA | 6 (40%) | 3 (8%) |  |
| IB | 3 (20%) | 7 (17%) |  |
| IIA | 1 (7%) | 4 (10%) |  |
| IIB | 1 (7%) | 17 (43%) |  |
| III | 2 (13%) | 9 (23%) |  |
| IV | 1 (7%) | 0 (0%) |  |
| Negative resection margin , n (%) | 12 (80%) | 32 (80%) | 1.000 |
| R, resectable; BR, borderline resectable; LA, locally advanced; M, metastasis; G, histological grade. Gemcitabine- and S-1-based chemotherapies with or without radiation for neoadjuvant treatment. **p*<0.05 by chi-square test. | | | |

| Supplementary Table 3. High- and low-grade regression groups based on ART scores in 42 chemoradiotherapy patients | | | |
| --- | --- | --- | --- |
|  | High-grade regression group  (ART score 0,1,2,3) | Low-grade regression group  (ART score 4) | P value |
| No. of patients | 17 | 25 |  |
| Age, years |  |  |  |
| Median (range) | 68 (44-76) | 68 (51-78) |  |
| ≥70 (%) | 7 (41%) | 11 (44%) | 0.856 |
| Sex, male (%) | 10 (59%) | 18 (72%) | 0.374 |
| Tumor location (%) |  |  |  |
| head/ body and tail | 8 (47%)/ 9 (53%) | 16 (64%)/ 9 (36%) | 0.276 |
| Preoperative diagnosis (%) |  |  |  |
| R/ BR/ LA/ M | 2 (12%)/ 14 (82%)/ 0 (0%)/ 1 (6%) | 1 (4%)/ 21 (84%)/ 3 (12%)/ 0 (0%) | 0.224 |
| Tumor differentiation, n (%) |  |  |  |
| G1/ G2/ G3/ others | 8 (47%)/ 5 (29%)/1 (6%)/ 3 (18%) | 12 (48%)/11 (44%)/2 (8%)/0 (0%) | 0.169 |
| Vascular invasion (%) |  |  |  |
| Negative/ Positive | 11 (65%)/ 6 (35%) | 4 (16%)/ 21 (84%) | 0.001* |
| Perineural invasion (%) |  |  |  |
| Negative/ Positive | 8 (47%)/ 9 (53%) | 4 (16%)/ 21 (84%) | 0.029* |
| Stage (UICC 8th) (%) |  |  |  |
| 0 | 0 (0%) | 0 (0%) | 0.182 |
| IA | 8 (47%) | 3 (12%) |  |
| IB | 5 (29%) | 12 (48%) |  |
| IIA | 0 (0%) | 1 (4%) |  |
| IIB | 4 (24%) | 7 (28%) |  |
| III | 1 (6%) | 1 (4%) |  |
| IV | 0 (0%) | 0 (0%) |  |
| Negative resection margin , n (%) | 15 (88%) | 19 (76%) | 0.982 |
| R, resectable; BR, borderline resectable; LA, locally advanced; M, metastasis; G, histological grade. Gemcitabine- and S-1-based chemotherapies with or without radiation for neoadjuvant treatment. **p*<0.05 by chi-square test. | | | |

| Supplementary Table 4. Association between each grading system and survival | | | | |
| --- | --- | --- | --- | --- |
|  | High-Grade Regression | Low-Grade Regression | P value for OS | P value for RFS |
| Evans', grade/n | I/ 71 | II, III, IV/ 26 | 0.786 | 0.783 |
|  | I, IIa/ 81 | IIb, III, IV/ 16 | 0.519 | 0.569 |
|  | I, II/ 93 | III, IV/ 4 | 0.382 | 0.283 |
|  | I, II, III/ 96 | IV/ 1 | 0.414 | 0.261 |
| CAP, grade/n | 0/ 1 | 1, 2, 3/ 96 | 0.414 | 0.261 |
|  | 0, 1/ 7 | 2, 3/ 90 | 0.296 | 0.133 |
|  | 0, 1, 2/ 52 | 3/ 45 | 0.147 | 0.120 |
| MDA, grade/n | 0/ 1 | 1, 2/ 96 | 0.414 | 0.261 |
|  | 0, 1/ 7 | 2/ 90 | 0.296 | 0.133 |
| JPS, grade/n | 1b, 2,3,4/ 75 | 1a/ 22 | 0.162 | 0.852 |
|  | 2,3,4/ 24 | 1/ 73 | 0.193 | 0.898 |
|  | 3, 4/ 5 | 1, 2/ 92 | 0.254 | 0.149 |
|  | 4/ 1 | 1, 2, 3/ 96 | 0.414 | 0.261 |
| ART, grade/n | 0/ 1 | 1, 2, 3, 4/ 96 | 0.414 | 0.261 |
|  | 0, 1/ 9 | 2, 3, 4/ 88 | 0.041* | 0.042* |
|  | 0, 1, 2/ 16 | 3, 4/ 81 | 0.007* | 0.014* |
|  | 0, 1, 2, 3/ 32 | 4/ 65 | 0.001* | 0.005* |
| OS, overall survival; RFS, recurrence-free survival; CAP, College of American Pathologists; MDA, MD Anderson; JPS, Japanese Pancreas Society; ART, Area of Residual Tumor. **p*<0.05 determined by the log-rank test. The groupings highlighted in gray indicate high- and low-grade regression groups as determined using ROC analysis and applied for survival analysis (as shown in Figure 3). | | | | |
